# Synthesis and Neuropharmacological Evaluation of Some Novel Quinoxaline 2, 3-Dione Derivatives

**DOI:** 10.1100/2012/718023

**Published:** 2012-05-02

**Authors:** Selvaraj Jubie, Rajamanickam Gayathri, Rajagopal Kalirajan

**Affiliations:** ^1^Department of Pharmaceutical Chemistry, JSS College of Pharmacy, Off Campus, JSS University, Mysore 570015, India; ^2^Department of Pharmaceutical Chemistry, JSS College of Pharmacy, Rocklands, Ooty 643001, India

## Abstract

Quinoxaline-2, 3-dione obtained from cyclocondensation reaction of o-phenylene diamine with oxalic acid was reacted with three different ketones and formaldehyde to give the corresponding Mannich bases in satisfactory yield. Their structures were confirmed by using ^1^H NMR, IR, and mass analysis. In pharmacological evaluation, the synthesized compounds showed its curative effect against ethidium-bromide-induced demyelination in rats. For the purpose, different screening methods such as open field exploratory behavior test, rota rod test, grip strength test, beam walk test, and photo actometer test were performed. Ethidium bromide induction showed muscle weakness; muscle discoordination; loss of locomotor activity, and so forth, the synthesized drugs reversed all the above-mentioned neuromuscular disorders caused by ethidium bromide administration.

## 1. Introduction

Quinoxaline-2, 3-dione derivatives are important classes of nitrogen-containing heterocycles, as they constitute useful intermediates in organic synthesis [1]. In particular, quinoxaline scaffolds were found as a core unit in a number of biologically active compounds. Quinoxaline including their fused ring derivatives display diverse pharmacological activities such as neuroprotective agents, antifungal, antibacterial, radio protective, anticonvulsant, antimalarial, anticancer, potent antithrombotic, analgesic, anti-inflammatory, antiglaucoma, antiparasite, antituberculosis, hypoglycemic, antiviral, anti-HIV, anthelmintic activities, antidepressant, NMDA receptor antagonist, and antimalarial activities [2–5]. Among the glutamate receptor antagonists, AMPA-R antagonists appear to be free from side effects such as schizophrenia and have shown effectiveness against neuronal death, even if administered after-ischemia. In consequence, AMPA-R antagonists have been reported to be effective in the therapy of neurodegenerative disorders such as ischemic stroke, epilepsy, head trauma, and Alzheimer's disease [6, 7].

 In the literature are described different series of AMPA receptor antagonistic, one of which is based on the quinoxaline-2, 3-dione structure, which have high affinity and selectivity. Typical examples are

1. 7-nitro-2, 3-dioxo-1, 2, 3, 4-tetrahydroquinoxaline-6-carbonitrile **(CNXQ)**,
2. 6-(1H-imidazol-1-yl)-7-nitro-1, 4-dihydroquinoxaline-2, 3-dione **(YM90K)**,
3. [7-(1H-imidazol-1-yl)-6-nitro-2,3-dioxo-3,4-dihydroquinoxaline-1(2H)-yl] acetic acid **(YM872)**,
4. 1-[4-(carboxymethyl)-7-nitro-2, 3-dioxo-1,2,3,4-tetraquinoxaline-6-yl]-1Hpyrrole-3-carboxylic acid **(Lu123313)**,
5. 6-nitro-2,3-dioxo-1,2,3,4-tetrahydrobenzo [f] quinoxaline-7-sulphonamide **(NBXQ)**,
6. 9-(1H–imidazol-1-yl)-8-nitropyrazolo [1,5-c]quinazoline-2,5(3H,6H)-dione (RO-48-8587).

**Scheme 1:**
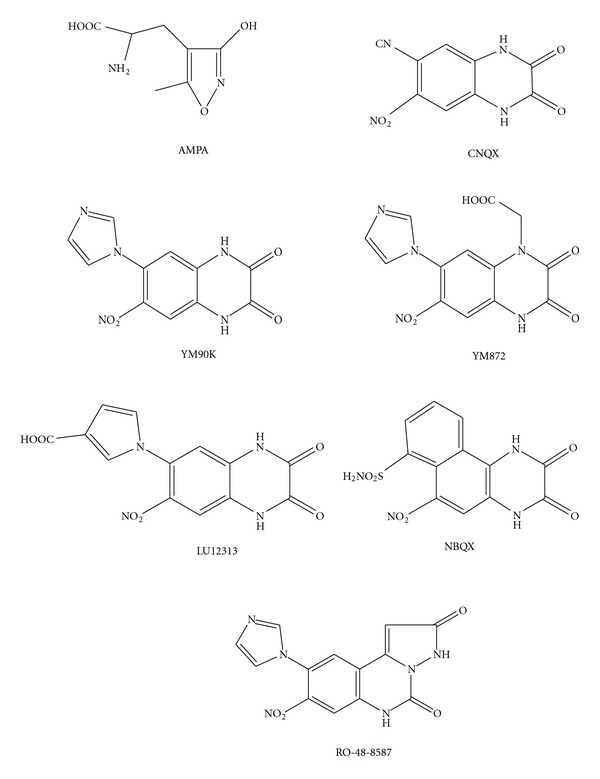
Different AMPA receptor antagonists.

These compounds are screened for neuropharmacological activity in mice and rats (analgesia, sedation, convulsion, anxiety, memory, and psychosis) (Scheme 1). 

The available AMPA antagonistic compounds such as **6-cyano-7-nitroquinoxaline-2, 3-dione (CNQX)**,** 6, 7-dinitroquinoxalone-2, 3-dione (DNQX)**, or earlier compounds such as **glutamic acid diethyl ester (GDEE) **had very little antagonist activity in vivo due to their lack of affinity.

Since these compounds are based on quinoxaline-2, 3-dione, we were planning to synthesize some novel quinoxaline dione derivatives and to evaluate their antagonistic action through neuroprotection.

## 2. Results and Discussion

### 2.1. Chemistry

**Scheme 2:**
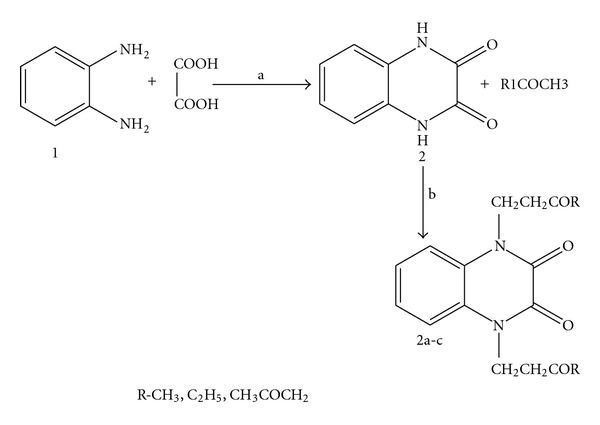
Synthesis of the compounds; reagents and conditions; (a) reflux on an oil bath, 1.5 h; (b) 40% HCHO, R_1_COCH_3_, DMF, stir, 6 h.

The synthetic route followed for obtaining compounds (2a–c) are outlined in Scheme 2. Thus, cyclocondensation of o-phenylene diamine (1) with oxalic acid in presence of hydrochloric acid afforded quinoxaline 2, 3-dione (2) by both conventional and microwave irradiation method. In microwave method, the percentage yield was higher than the conventional method. The NH groups of compound 2 were undergone Mannich reaction with different ketones, and formaldehyde afforded the Mannich bases (2a–c). The percentage yield was 50–85%. The synthesized compounds were checked for their purity through melting point determination and TLC. Further, all the compounds were characterized by spectral analyses such as IR, ^1^H NMR, and mass spectra. The data are consistent with the assigned structures.

### 2.2. Pharmacology

The synthesized compounds, compounds **2a**, **2b**, and **2c** were screened for neuroprotective activity. Before induction of demyelinating lesions to the animals, acute toxicity study was carried out to find out the doses. Most demyelination of CNS produces difficulty in loco motor and behavioral pattern. Symptoms are confusion, muscle weakness, numbness, muscle discoordination, and hind limb paralysis. Hence, the following animal tests were carried out to assess the introduction and to evaluate the remyelination in animals. The tests are

- open-field exploratory behavior test,
- rotarod test,
- gripstrength test,
- beam walk test,
- photoactometer test.

The animals subjected for the above tests were observed for four weeks except for open-field exploratory behaviour test, which was carried out for only one week. The results obtained are as follows.

#### 2.2.1. Open-Field Exploratory Test

From this study, it was found that ethidium-bromide-treated group showed decreased locomotor activity at the end of the first week. Among the synthesized quinoxaline-2, 3-dione, **2c** showed the highest activity with increased in ambulation and decreased freezing time with that of ethidium-bromide-induced groups. It indicated that due to the induction of dose of the synthesized compounds, there was an alteration in ethidium-bromide-induced toxic locomotor effect. The results are given in Table 1.

#### 2.2.2. Grip Strength Test

The experiment was carried out to measure the muscle coordination and strength, when the animals were put in the wire mesh. The time taken to catch the wire and the time spent on the mesh by holding the wire were measured. Ethidium-bromide-treated animals took more time to catch the wire with their hind legs, and the time spent on the wire by holding position was also was very little. These results clearly indicate the administration of the toxic ethidium bromide resulted in decreased muscle coordination and grip strength. It may be due to demyelination of nerve fibres leading to the impaired conduction of nerve impulses. Treatment of animals with **2a**, **2b**, and **2c** reversed the observed grip strength deficit. The results are given in Table 2.

#### 2.2.3. Rota Rod Test

To analyse the muscle coordination, rota rod test was performed. The results indicated the ethidium bromide treated rats took very short time to fall from the rota rod apparatus, indicating the loss of muscle coordination. At the end of the fourth week, rats treated with the synthesized compounds spent increased time on the rota rod, indicating the muscle coordination had been recovered as that of the control group. The synthesized compounds showed little higher effects at the end of fourth week than standard drug. The results are given in Table 3.

#### 2.2.4. Beam Walk Test

This is used to evaluate the activity of the drugs interfering with motor coordination. In this test, the ability of the animal to walk on the beam was evaluated. It had been observed that ethidium bromide treated animals walked less distance and falling time was also less comparable to control group.

Rats treated with the compounds **2a**, **2b**, and **2c**, at the end of fourth week, increased distance of walk on the beam and falling time, indicating the muscle coordination had been recovered as that of the control group. The synthesized compounds showed moderate activity when comparable to the standard. The results are given in Table 4.

#### 2.2.5. Photoactometer Test

To study the locomotor activity (CNS-depressant activity) of ethidium bromide and its recovery by means of synthesized drugs on rats, photoactometer was mainly used. The animals subjected for this test were observed for the 1st, 2nd, and 4th week. The results clearly indicated that the ethidium-bromide-treated rats moved lesser square-shaped space of the instrument at the end of the 1st week, and the same was maintained at the end of four-week period.

Treatment of animals with the selected doses of synthesized drugs in the above tests significantly increased the observed locomotor activity which was comparable to that of the control group. The compounds 2a, 2b, and 2c showed greater activity than the standard. The results are given in Table 5.

### 2.3. Histopathological Studies

**Figure 1:**
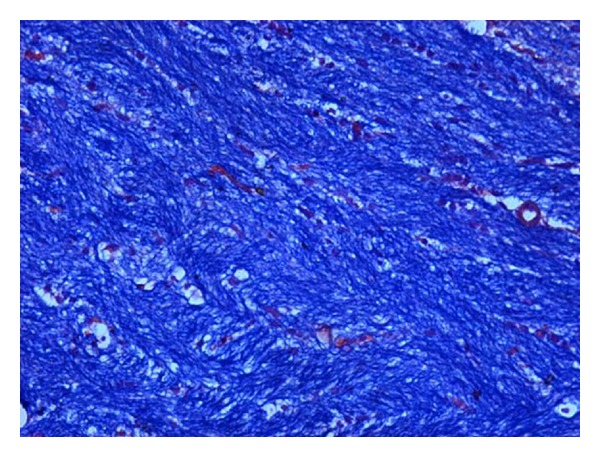
High-magnification photomicrographs of LFB-stained midbrain sections from normal control group.

The demyelination induced by ethidium bromide on rats and remyelination by synthesized drugs were confirmed by the histopathological studies. The midbrain section of the brain was stained with Luxol fast blue to study the neurodegeneration effects of EB and protective effect of standard and synthesized drugs 2a, 2b, and 2c in the midbrain region. The effect of ethidium bromide, dose of 10 *μ*L solution of 0.1% ethidium bromide and synthesized drugs in the dose of 20 mg/kg body weight on rats were shown (Figures 1 and 6). The myelinated fibres were visualized as blue in colour, neutrophils in pink, and nerve cells in purple.

**Figure 2:**
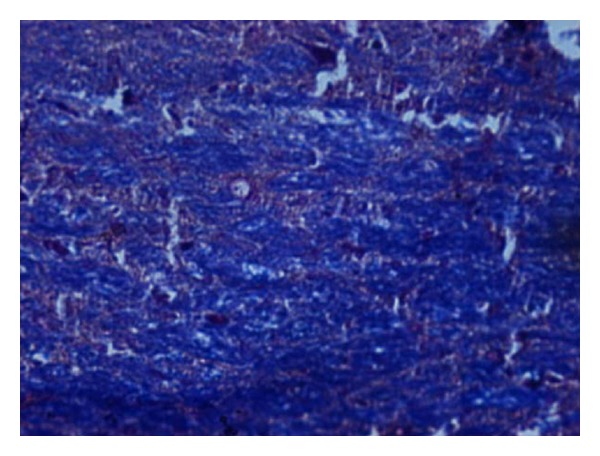
High-magnification photomicrographs of LFB-stained midbrain sections from ethidium bromide induced group (negative control).

**Figure 3:**
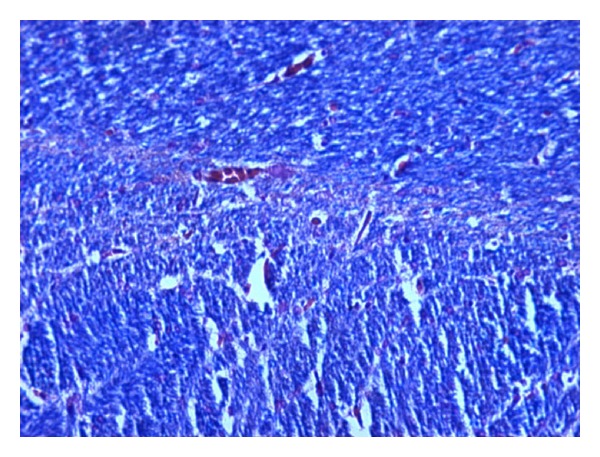
High-magnification photomicrographs of LFB-stained midbrain sections from standard drug-treated group.

**Figure 4:**
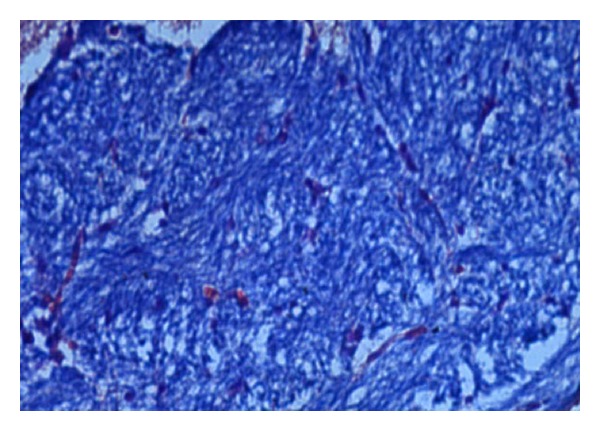
High-magnification photomicrographs of LFB-stained midbrain sections from 4b-treated group.

**Figure 5:**
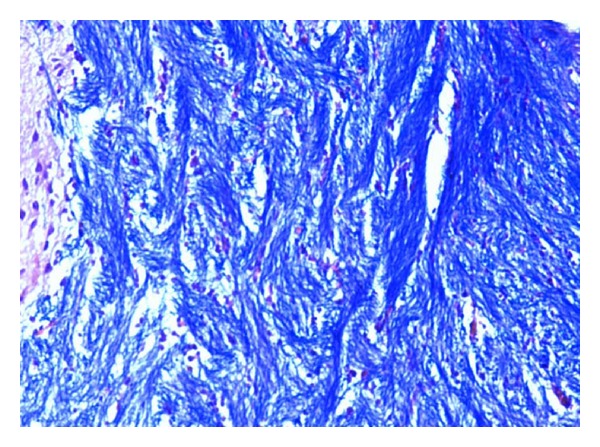
High-magnification photomicrographs of LFB-stained midbrain sections from 5b-treated group.

**Figure 6:**
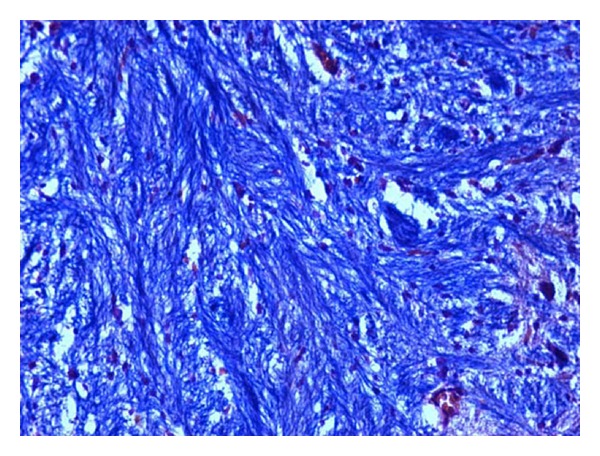
High-magnification photomicrographs of LFB-stained midbrain sections from 5b-treated group.

The midbrain regions of control rats were found to be intact, and no destruction of the myelin sheath was observed (Figure 1). There is loss of myelinated fibres in the EB induced rats (Figure 2). It demonstrated distinct degeneration of myelin sheath indicating neurotoxicity. Administration of standard drug and synthesized drugs (2a, 2b, and 2c) on rats accelerated the myelin regeneration (Figures 3, 4, 5 and 6).

## 3. Conclusion

In pharmacological evaluation, the selected synthesized drugs showed their curative effect against intracranially administered ethidium-bromide-induced demyelination in rats. For the purpose, different screening methods such as open-field exploratory behavior test, rota rod test, grip strength test, beam walk test, and photoactometer test were performed. Ethidium-bromide-induction showed muscle weakness; muscle in coordination; loss of loco motor activity, and so forth the selected synthesized drugs reversed all the above said disorders developed as a result of ethidium-bromide-induction.

Hence from the above results, it was established that the selected synthesized drugs are useful to counteract different demyelinating disorders such as multiple sclerosis (together with the similar diseases called idiopathic inflammatory demyelinating diseases), tranversesmyelitis, Devic's disease, progressive multifocal leukoencephalopathy, optic neuritis, Guillain-Barre syndrome and its chronic counterpart, chronic inflammatory demyelinating polyneuropathy, and so forth which will be a great relief for our society to overcome the above-said neurological disorders. The current paper offers compelling, but perhaps not conclusive, arguments for an association between behavioral and brain anatomical changes induced by EB and its attenuation by selected synthesized quinoxaline dione derivatives.

### 3.1. Experimental

All the solvents and materials were reagent-graded and purified as required. Melting points were taken in open glass capillary using Veego VMP-1 melting point apparatus and are uncorrected. IR spectra were recorded on Shimadzu FT-IR spectrometer model using KBr discs. The NMR spectra (DMSO-d_6_) were recorded on Bruker DRX-300 spectrometer with TMS as an internal standard. The mass spectra were measured on a Shimadzu LCMS 2010A spectrometer.

### 3.2. Synthesis of Quinoxaline-2, 3-Dione (2) under Thermal Conditions

A powdered mixture of oxalic acid dihydrate (0.01 mole, 1.26 g) and o-phenylene diamine (0.01 mole, 1.0814 g) was refluxed by using an oil bath for 1.5 hours and cooled. The product that separated was filtered and washed with water and recrystallized with 5% NaOH/dil HCl to give the colourless crystals.

#### 3.2.1. Under Microwave Irradiation

A powdered mixture of oxalic acid dihydrate (0.01 mole, 1.26 g) and o-phenylene diamine (0.01 mole, 1.0814 g) was put in an open beaker, and 1 mL of water added and mixed thoroughly. The mixture was irradiated in a catalyst microwave system at an emitted power of 400 W for 3 min. 100 mL of water was added, followed by further irradiation for 1 min to give a clear solution and then left to stand at room temperature. The product obtained was filtered, washed with water, and crystallized with 5% NaOH/dil HCl to give colourless crystals **2**, (88%), mp > 340°C; R_f_  (chloroform/methanol 9 : 1) 0.52; IR (KBr): 1708.9 and 1681 (C=O), 1247 (C–N), 3344 (N–H), 854 (Ar C–H), 1593 (C=C); ^1^H NMR (DMSO-d_6,_300 MHZ): *δ* 11.7 (s, **2H**, NH), 7 (m, **4H**, Ar-H); ms (m/z): M+ calculated 162.15, found 161.98.

### 3.3. General Procedure for the Synthesis of Mannich Bases (2a–c)

A mixture of quinoxaline-2, 3-dione (0.01 mol, 0.162 gm) in DMF, formaldehyde (40%, 1.5 mL) and different ketones (0.02 mol) was stirred at room temperature for 6 h. The precipitated solid was filtered under suction, washed with ethanol and recrystallized from hot ethanol, to give colourless crystals **2a**–**c**.

#### 3.3.1. 1,4-Bis(3-Oxobutyl) Quinoxaline-2,3(1H,4H)-Dione (2a)

Colourless crystals 2a, (70%), mp > 300°C; R_f_  (benzene/acetone 9 : 1) 0.23; IR (KBr): 1708.9 and 1681 (C=O), 1247.9 (C–N), 1473 (CH_2_); ^1^H NMR (DMSO-d_6, _300 MHZ): *δ* 2.1 (s, **6H**, CH3), 3.3 (m, **4H**, N–CH_2_–CH_2_), 6.5–7 (m, **4H**, Ar–H); ms (m/z): M+ calculated 302.15, found 301.98.

### 3.4. Ethidium-Bromide-Induced Model 

Healthy, adult Wister rats of both sex male: female (1 : 1) weighing 120–140 gm were used which was approved by the Institutional ethical committee. The animal house was well ventilated, and animals were kept at 12 ± 2°C. The animals were caged in large specious hygienic cages during the course of experimental method. The animals were fed with rat pallet feed, supplied by Hindustan Liver Pvt. Ltd. The place of experiment to be conducted was kept in very hygienic condition.

This chemical is mainly used for local demyelination. Here, demyelinating lesions were created in brain. For the surgical procedure, the animals from both treatments were anesthetized with ketamine chloridate and xylazine (5 : 1; 0.1 mL/100gm), and after saving the fronto-parietal-occipital area, antisepsis with 2% iodine solution was carried out. With the aid of a roof motor of orthodontic use and a drill number 2, a hole was made 0.85 cm to the right of the bregma until exposing the durameter. With the use of a Hamilton syringe with a removable needle of caliber 26 s, the solutions were injected in the cistern pontis (basal), an enlargement of the subarachnoid space on the ventral surface of the pons. Ten microlitres of EB was injected to the animals from groups II to VII, and the same volume of 0.9% saline solution was injected to the animals from group I. The durameter was left open and the skin, together with the remainder of the subcutaneous tissue, was sutured with a nylon thread 4.0 [8].

For the evaluation of comparative protective effect of synthesized drugs, selected doses were administered to animals for 28 days after the inoculation of ethidium bromide.

The experimental design of the investigation was carried out in seven groups among which the Gr-I and Gr-II were having three animals each and from Gr-III to Gr-VII the number of animals was five. The groupings were as follows:

**Group I (three animals)**

Served as solvent control which received only 0.3% CMC (2 mL) orally and antioxidant in 0.3% CMC solution (2 mL) once orally daily.

**Group II (three animals)**

Received demyelinating agent ethidium bromide (10 *μ*L solution of 0.1% ethidium bromide in PBS) intracranially only once and 0.3% CMC solution (2 mL) once orally daily.

**Group III (five animals)**

Received demyelinating agent ethidium bromide (10 *μ*L solution of 0.1% ethidium bromide in PBS) intracranially only once and antioxidant in 0.3% CMC solution (2 mL) once orally daily.

**Group IV (five animals)**

Received demyelinating agent ethidium bromide (10 *μ*L solution of 0.1% ethidium bromide in PBS) intracranially only once and 2,3-dioxo-1,2,3,4-tetrahydroquinoxaline-6-sulphonyl (2-methyl) benzimidazole in 0.3% CMC solution (2 mL) once orally daily.

**Group V (five animals)**

Received demyelinating agent ethidium bromide (10 *μ*L solution of 0.1% ethidium bromide in PBS) intracranially only once and quinoxaline-2, 3-bis phenyl hydrazone 0.3% CMC solution (2 mL) once orally daily.

**Group VI (five animals)**

Received demyelinating agent ethidium bromide (10 *μ*L solution of 0.1% ethidium bromide in PBS) intracranially only once and 1, 4-bis (3-oxobutyl) quinoxaline-2, 3(1H, 4H)-dione in 0.3% CMC solution (2 mL) once orally daily.

#### 3.4.1. Methods of Testing

**(i) Open-Field Exploratory Behavior Test**

This test has been utilized for behavioral changes in rodents to a novel environment and has been used to detect anxiogenic and anxiolytic activity under identical situation. A typical apparatus suitable for rats comprises of large black area (96 × 96) cm with height 96 cm walls. The floor is divided into 16 squares by white lines. The apparatus is placed in a dim light room. The rats will be placed in a corner of the apparatus individually and observed for 15–20 min. The parameters are as follows.

- Ambulation: number of squares crossed by the animal in the apparatus.
- Freeze: Periods of immobility, the time for which the animal shows no movement.
- Rearing: number of times when animal stands on its rear paws.
- Defecation: number of fecal pellets.

Since exposure to a novel environment is associated with “emotionality”, an anxious animal is one which shows reduced ambulation associated with periodic “freeze” and reduced normal behavior like growing, concomitant with increased autonomic activity resulting in increase defecation and urination accentuated by anxiogenic and anxiolytic agent. Each animal was placed individually at one corner of the apparatus and watched the above parameters for 15–20 minutes [9].

**(ii) Grip Strength Test**

This test is performed to access muscular strength or neuromuscular function in rats which will be influenced not only by sedative drugs and skeletal muscle relaxant compounds but also by toxic agents. The method was discovered as “test de” grippment by Boissler and Simon (1960). The animals will be proposed to a horizontal thin thread or metallic wire suspended about 10 cm in the air, which they immediately grasp with the fore limbs. Normal animals are able to catch the wire with hind limbs and to climb up with in 5 sec. The animals that are not able to touch the wire are considered as impaired. Each rat was tested for grip strength. Parameters observed were the time required to catch the wire with hind limb, time of falling, time of immobility, and number of fecal pellets. The tests were carried out for 5 min for each animal [10].

**(iii) Rota Rod Method**

This test is used to evaluate the activity of drugs interfering with motor coordination. In 1956, Paunam and Mixa suggested that the skeletal muscle relaxant-induced by test compound can be evaluated by testing the ability of rat to remain on a revolving rod. The application consists of horizontal metal rod of 3 cm diameter attached to a motor with the speed 20–25 rpm. The rod is divided in five reactions with wooden compartment. It allows simultaneous testing of five rats. The rod is in height of 50 cm above the table top in order to discourage the animal from falling off. The compartment made with wooden cardboard to restrict the escape of animals when they fall from the rod. The test animals along with normal animals will be tested for the time of falling from the roller, number of fecal pellets and its behavior. The rats were placed on rotating rod, and observations were made for the falling time, number of fecal pellets, frequency of urination and its behaviour [11].

**(iv) Beam Walk Test**

This test is used to evaluate the activity of the drugs interfering with motor coordination. In this test, the ability of animal to walk on the beam is evaluated. The apparatus consists of a horizontal metal rod of 1 cm diameter which is evaporated by two-side stands at 30 cm height. The animals were kept in the center of the rod to allow walking on the beam. The parameters such as the number of fecal pellets, distance of walk, time of immobility, and falling time are evaluated. The animals were observed for 5 min. Each animal was placed in the center of the metal beam and observed for five minutes for the parameters such as fall of time, distance of walk, number of fecal pellets, freezing time and its behaviour [11].

**(v) Photoactometer Test**

To study the locomotor activity (CNS-depressant activity) of ethidium bromide and its recovery by means of quinoxaline dione derivatives on rats, photoactometer is mainly used. Most of the CNS-acting drugs influence the loco motor activities in human and animals. Demyelinating agents such as ethidium bromide act as CNS depressant which decreases the loco motor activities. In other words, loco motor activity can be an index of wakefulness (alertness) of mental activity. The loco motor activity (horizontal activity) can be easily measured using an actophotometer which operates on photoelectric cell which is connected in a circuit with a connector. When the beam of light falling on the photo cell is cut off by the animal, a count is recorded. An actophotometer may have square or round area in which the animal moves. Both mice and rat can be used for testing in the apparatus. The total number of cutoff was measured mechanically for ten minutes per animal, and observations were made.

The animals subjected for the above tests will be observed on the 1st, 2nd, and 4th week, but open-field exploratory behaviour test observation will be made only for the 1st week.

#### 3.4.2. Histopathological Studies

To confirm the demyelination used in the model, three rats injected with saline (group I), three rats injected with EB alone (group II), and three rats from treated groups III to VII were used for histological analysis of the lesion. The rats were perfused under deep anesthesia with 10% buffered formaline via the left ventricle at the end of the behavioural studies. Brain stem coronal slices with the lesion were embedded in paraffin for routine processing, and 10 *μ*m sections were produced and stained with Luxol fast blue staining [12].

## Figures and Tables

**Table 1:** Open-field exploratory test.

Groups	Ambulation	Rearing	Fecal pellets	Freezing time
Control	133.7 ± 0.6667	19.53 ± 0.2186	0.7 ± 0.3930	51.53 ± 0.1453
Ethidium				
Bromide	87.67 ± 6.960	13.0 ± 1.155*	3 ± 0.5774*	68.53 ± 0.2028
Antioxidant	108.2 ± 2.083***	25.2 ± 1.530**	3.8 ± 0.2000**	58.48 ± 0.675***
Comp2a	115.8 ± 0.3742**	26.4 ± 0.050**	3.2 ± 0.489	40.50 ± 0.5117***
Comp2b	109.4 ± 0.335***	33.6 ± 0.743***	3 ± 0.5477**	43.46 ± 0.1536***
Comp2c	153.4 ± 0.6000***	39.42 ± 0.159***	3.6 ± 0.6782**	46.28 ± 0.1020***

*P* < 0.05; ***extremely significant; **moderately significant; *significant.

**Table 2:** Grip strength test.

Groups	Time taken to catch the wire with hind limb	Total time the animal catches the wire	Falling time	Fecal pellets
Control	8.567 ± 0.0333	186.7 ± 0.028	92.6 ± 0.881	1 ± 0
Ethidium				
Bromide	4.3 ± 0.100	143.0 ± 0.603***	124.7 ± 0.480***	4.3 ± 0.333***
Antioxidant	3.740 ± 0.0748***	154 ± 1.140***	104.8 ± 0.985***	3 ± 0.3162**
Comp2a	3.320 ± 0.2354**	151.2 ± 0.274**	99.20 ± 0.020*	2.6 ± 0.2449*
Comp2b	3.2 ± 0.05477***	148.4 ± 0.707***	105 ± 0.707*	4.750 ± 0.478**
Comp2c	1.68 ± 0.1068**	161.4 ± 0.534*	101.8 ± 0.916**	3.4 ± 0.4***

*P* < 0.05; ***extremely significant; **moderately significant; *significant.

**Table 3:** Rota rod test.

Groups	Falling time	Fecal pellets
Control	311.7 ± 0.6667	4 ± 0.5774
Ethidium		
Bromide	113.7 ± 0.8819***	1.6 ± 0.333***
Antioxidant	188 ± 1.095***	2.2 ± 0.2***
Comp2a	203.2 ± 0.9695***	1.8 ± 0.2***
Comp2b	177.2 ± 1.562***	2.8 ± 0.3742*
Comp2c	212.2 ± 0.9695***	1.8 ± 0.2***

*P* < 0.05; ***extremely significant; **moderately significant; *significant.

**Table 4:** Beam walk test.

Groups	Distance walked	Total time taken to walk	Falling time	Fecal pellets
Control	209.5 ± 0.2082	300	127.6 ± 0.057	1 ± 0
Ethidium				
Bromide	35.47 ± 0.2906	300	166.5 ± 0.29**	5.76 ± 0.39**
Antioxidant	171.6 ± 0.7928***	300	142.8 ± 0.58**	1.6 ± 0.24***
Comp2a	142.5 ± 0.1435***	300	111.4 ± 0.170*	1.4 ± 0.24***
Comp2b	158.4 ± 0.147***	300	92.3 ± 0.803***	1.2 ± 0.2***
Comp2c	137.4 ± 0.12.8***	300	118.3 ± 0.0816***	1.6 ± 0.24***

*P* < 0.05; ***extremely significant; **moderately significant; *significant.

**Table 5:** Photoactometer test.

Groups	Time of movement (sec)	Counts of movements
Control	600	283.3 ± 1.202
Ethidium bromide	600	149.0 ± 2.082***
Antioxidant	600	215.2 ± 1.2***
Comp 2a	600	257.2 ± 1.158***
Comp2b	600	249.1 ± 0.678***
Comp2c	600	232.8 ± 1.241***

*P* < 0.05; ***extremely significant; **moderately significant; *significant.
